# Copy number variation in livestock: A mini review

**DOI:** 10.14202/vetworld.2018.535-541

**Published:** 2018-04-26

**Authors:** V. Bhanuprakash, Supriya Chhotaray, D. R. Pruthviraj, Chandrakanta Rawat, A. Karthikeyan, Manjit Panigrahi

**Affiliations:** Division of Animal Genetics, ICAR - Indian Veterinary Research Institute, Izatnagar, Bareilly - 243122, Uttar Pradesh, India

**Keywords:** copy number variation, copy number variation regions, livestock, single nucleotide polymorphisms, quantitative trait loci

## Abstract

Copy number variation (CNV) is a phenomenon in which sections of the genome, ranging from one kilo base pair (Kb) to several million base pairs (Mb), are repeated and the number of repeats vary between the individuals in a population. It is an important source of genetic variation in an individual which is now being utilized rather than single nucleotide polymorphisms (SNPs), as it covers the more genomic region. CNVs alter the gene expression and change the phenotype of an individual due to deletion and duplication of genes in the copy number variation regions (CNVRs). Earlier, researchers extensively utilized SNPs as the main source of genetic variation. But now, the focus is on identification of CNVs associated with complex traits. With the recent advances and reduction in the cost of sequencing, arrays are developed for genotyping which cover the maximum number of SNPs at a time that can be used for detection of CNVRs and underlying quantitative trait loci (QTL) for the complex traits to accelerate genetic improvement. CNV studies are also being carried out to understand the evolutionary mechanism in the domestication of livestock and their adaptation to the different environmental conditions. The main aim of the study is to review the available data on CNV and its role in genetic variation among the livestock.

## Introduction

The copy number variants (CNVs) are a structural variation in the genome of an individual in the form of losses or gains of DNA fragments. CNV is an important source of genetic and phenotypic variation [1]. Union of overlapping CNVs detected in two different samples are called copy number variation regions (CNVRs) [2]. The difference in the copy number of CNVR genes results in changes in the gene expression and phenotypic variation due to altering gene dosage and gene disruption effect by the deletion, duplication, inversions, and translocations of the gene. It is a source for evolutionary mechanisms [3]. If CNV exists in the protein coding region, it alters the protein function, whereas in the regulatory region, it alters the gene expression level [4]. The current review helps in understanding the CNV and its role in the improvement of economic traits in livestock.

## Mechanisms of CNV Formation

Non-allelic homologous recombination (NAHR), non-homologous end-joining (NHEJ), fork stalling and template switching (FoSTeS), and L1-mediated retro transposition are some of the mechanisms which generate rearrangements in the genome and possibly account for the majority of CNV formation [5,6]. NAHR occurs in meiosis and mitosis due to recombination between the two regions of similar sequence between the non-homologous chromosomes. If crossing over occurs between the sister chromatids, it can increase the segment of DNA at the expense of another which may result in duplication, deletion, and inversion of the segment of chromosome. NHEJ mechanism is utilized by cells to repair DNA double-strand breaks (DSBs) caused by ionizing radiation or reactive oxygen species and physiological forms of DSBs such as variable (diversity) joining (V(D)J) recombination [7,8]. FoSTeS is a DNA replication-based mechanism which can account for Complex Genomic Rearrangements and CNVs [9]. L1 transposition occurs through reverse transcription and integration [10]. A number of studies have been carried out to identify the CNV in different species such as cattle [11-14], sheep [15,16], goat [17], pig [18-21] and chicken [22].

## Algorithm used for Identification of CNV

SNP arrays are being used normally for CNV detection and analysis in humans because of its availability and economic feasibility [2]. In general, most of the studies reported in literature for CNV detection in the population study used comparative genomic hybridization (CGH) arrays and SNP genotyping arrays [23]. Now a days, CNV detection and analysis by whole genome sequencing is practically possible due to decreased cost for next-generation sequencing (NGS) techniques. Relatively, sequencing has high resolution over genotyping as it covers the entire genome [24]. SNP arrays utilize a Log R ratio (LRR) and B allele frequency (BAF) which represents the copy numbers and allelic status of the population [25]. Large CNVRs are mostly identified with the SNP50 array since it lacks non-polymorphic probes. Multiple algorithms have been used to identify CNVs and CNVRs [26-29].

## PennCNV Software

It is a Hidden Markov Model (HMM) algorithm which integrates multiple parameters such as LRR, BAF, the population frequency of the B allele (PFB) of SNPs, the distance between neighboring SNPs and the allele frequency of SNPs [25-27]. It is based on fitting regression models with GC content to overcome genomic waves [30]. It improves the call rate and accuracy of boundary mapping by considering the pedigree information [12].

## CnvPartition

CnvPartition is based on a different proprietary sliding window approach which detects CNVs by processing LRR and BAF. Only those homozygous deletion events segregating in different animals were reported by this algorithm due to concern quality calls [31].

## cn.MOPS Algorithm

The cn.MOPS (Mixture of PoissonS) algorithm is based on the Bayesian approach for the detection of CNV in multiple samples for NGS data. It decomposes read variations across multiple samples into integer copy numbers and noise by its mixture components and Poisson distributions, respectively. The advantages of using this method are- it identifies overlapping sequences and estimates allele-specific copy numbers [32].

## QuantiSNP

QuantiSNP uses different HMMs unlike PennCNV. QuantiSNP uses both LRR and BAF frequency independently whereas in pennCNV treat them as combined. It uses a fixed rate of heterozygosity for each SNP [26].

## CNVFinder

It is a python package for CNV detection on whole exome sequencing data from amplicon-based enrichment technologies. This program uses SDe termed as experimental variability, in the LRR distribution [33].

## CNV Identification Studies in Domestic Species

### Cattle

Upadhyay *et al*. [34] using the Illumina BovineHD Genotyping in cattle identified 9944 CNVs and 923 CNVRs with a length of 61.06 Mb covering 2.5% of the bovine autosomes. These CNVRs were found to be associated with the quantitative trait loci which affect production traits, body measurements, and parasite resistance [23,35,36]. Incidence of overlap of CNVs reported among *taurine* cattle is higher than the overlap between *taurine* and *indicine* cattle. Largest CNV diversity was reported among the zebu cattle [37].

Recent studies reported that CNVs evolved 2.5 folds faster than SNPs and helped to promote a better adaptation in different environments [21]. Liu *et al*. [4] reported the high CNV abundance in *indicine* and African *taurine* cattle breeds than in European *taurine* using Vst for population differentiation which indicates the breed divergence and population history. Pezer *et al*. [38] suggested the variation in the CNV number may be due to the difference in effective population size, gene flow, and selection process among different populations. Upadhyay *et al*. [34] in their study reported that small populations might cause an increase in the CNVs, particularly deletion in CNVs. The discrepancy between the studies observed is due to the small sample size within the breed, large samples from multiple breeds and different SNP arrays used in the study [39]. Different studies using the same method and algorithm for the detection of CNVs, reported varying overlaps. The inconsistency of this overlaps between the studies is due to the platforms and algorithms of CNV calling, differences in size, and population structure under investigation.

Hou *et al*. [36] reported that the more CNV events were detected in *indicine* than in African groups and *taurine* breeds. This observation may suggest the independent domestication events of cattle in Europe, Africa, and Southeast Asia [40,41]. Hou *et al*. [36] using array CGH technique identified 25 germline CNVs in three Holstein bulls. The same group identified over 200 CNVRs from diverse cattle breeds. Fadista *et al*. [42] identified 304 CNVRs with a length of 15.8 Mb of the genome from 20 animals of 4 cattle breeds [36]. Bae *et al*. [11] also identified 368 unique CNV regions from 265 Korean Hanwoo cattle based on 50K SNP array covering 15.8Mb of the genome using PennCNV algorithm without considering the pedigree information and genomic waves. Recent studies on CNV detection using different approaches are given in the Table-1 [11-14,23,30,34-37,42-46] for cattle and Table-2 [14,16,47-52] for other species.

**Table-1:** CNVs detection studies in cattle using different approaches.

Author	Sample size	Breed	Method	CNVs	CNVRs	Total length (Mb)
Bae et al. [11]	265	1	50K	855	368	63.1
Hou et al. [12]	539	21	50K	3666	743	15.8
Jiang et al. [13]	2047	1	50K	219^a^ 169^b^ 140^c^	101	23.8
Wang et al. [14]	492	1	50K		389	70.4
Zhang et al. [23]	14	29	CGH		605	2.45
Bickhart et al. [30]	6	3	WGS		1265	55.6
Upadhyay et al. [34]		38	770K	9944	196	61.1
Xu et al. [35]	300	8	770K		257	12.4
Hou et al. [36]	674	27	770K	34311	3438	147
Da Silva et al. [37]	1717	1	770K	68007	7319	15.6
Fadista et al. [42]	20	4	CGH		254	15.8
Jiang et al. [43]	96	1	770K	1733	357	34.4
Sasaki et al. [44]	1481	1	770K	55593	861	43.6
Liu et al. [45]	20	17	CGH		200	36.2
Stothard et al. [46]	2	2	WGS		790	3.3

CNVRs identified by the different algorithm: Superscript a-PennCNV, b-GADA (Genome Alteration Detection Algorithm) and c-cnvPartition. CNV=Copy number variation, CGH=Comparative genomic hybridization, CNVRs=Copy number variation regions

**Table-2:** CNVs detection studies in domestic animals.

Species	Breed	CNVRs	Length (Mb)	Method	References
Pig	13	49	3131	WGS	Wang et al. [14]
Sheep	68	619	197	OvineSNP50 assay	Yang et al. [16]
Sheep	11	135	77.6	Bovine 385KaCGH arrays	Iafrate et al. [47]
Sheep	48	1296	121.8	OvineHD 600 K SNP array	Ma et al. [48]
Sheep	3	238	60.35	OvineSNP50 assay	Liu et al. [49]
Goat	10	127	90.3	Bovine 385KaCGH arrays	Hutt et al. [50]
Pig	55	49	754.6	Porcine SNP60 Beadchip	Kijas et al. [51]
Pig	2	172	80.41	PorcineSNP60	Xie et al. [52]

CNV=Copy number variation, CGH=Comparative genomic hybridization, CNVRs=Copy number variation regions, SNP=Single nucleotide polymorphisms

### Sheep

Yang *et al*. [16] identified 619 CNV regions, covering 197 Mb, corresponding to ~6.9% of the sheep genome and found several important CNV overlapping genes (*BTG3*, *PTGS1*, and *PSPH*) which are involved in the fetal muscle development, prostaglandin (PG) synthesis, and bone color. Ma *et al*. [48] identified 111 CNVRs from 160 Chinese sheep breeds covering 13.75 Mb of the sheep genome sequence. Fontanesi *et al*. [15] using a cattle–sheep 385K aCGH identified 135 CNV regions covering ~10.5 Mb of the sheep genome with reference to the bovine genome. It may suggest that several chromosome regions have a repetitive sequence of CNVRs between the species. CNVR overlapping with the homeobox transcription factor DLX3 was found to be associated with curly hair in sheep [48].

### Goat

Fontanesi *et al*. [17] identified 127 CNVRs covering about 11.47 Mb of the goat genome with reference to the bovine genome. Genes with environmental functions were over-represented in goat CNVRs as reported in other mammals [17]. Difference in the copy number at Agouti locus in sheep and goats contributes to the variability of coat color [53].

### Horse

Copy number variants account for about 1-3% of the horse genome and mostly of intragenic than those located in intergenic regions [54]. Ghosh *et al*. [55] using 400K WG tiling oligo array identified 258 CNV regions (CNVRs) comprised of 1.3% of the horse genome across all chromosome except chrY in 16 diverse breeds of horse and also found 20% of the identified CNVRs were located in intergenic regions.

### Chicken

Chicken has a unique genome arrangement due to the presence of micro- and macro-chromosome [56]. Griffin *et al*. [57] first studied the chicken CNV with aCGH to establish interspecies genomic rearrangement and they showed that there are more CNVs that involve coding genes than the non-coding sequences. Studies reported the phenotypic association of CNV in chickens, which include a pea-comb, late-feathering, dark brown plumage color, and dermal hyperpigmentation [16,58]. Recent studies for CNVs detection in chicken by different approaches are given in Table-3 [22, 59-66].

**Table-3:** CNVs detection studies in chicken using different approaches

Author	Sample size	Method	CNVs	CNVRs	Percentage coverage	Total length (Mb)
Crooijmans et al. [22]	64	aCGH	3154	1556	5.4	60
Zhang et al. [59]	475	60K SNP array	438^a^	271^a^	3.92^a^	40.26^a^
			291^b^	188^b^	2.98^b^	30.60^b^
Jia et al. [60]	746	60K SNP array	818	209	1.42	13.55
Yi et al. [61]	12	WGS	-	8840	9.4	98.2
Han et al. [62]	10	385 (aCGH) Genome array		281	1.07	12
Rao et al. [63]	554	60K SNP array	1875	383	3.97	41
Gorla et al. [64]	256	600K SNP Array	1924	1216	5.12	47
Strillacci et al. [65]	96	580K SNP array	1003	564	1.03	9.43
Fan et al. [66]	2	WGS	-	8839	24.6	

a Lean lines,

b Fat lines in chicken. CNV=Copy number variation, CGH=Comparative genomic hybridization, CNVRs=Copy number variation regions, SNP=Single nucleotide polymorphisms

## CNVRs genes and gene ontology (GO)

Bickhart *et al*. [30] reported the duplication of cathelicidin genes (*CATHL4*) in the Nellore cattle sample, but these genes were found only in a single copy in human and mice. CNV overlapping with *KIT* gene was found to be associated with color-sidedness in English Longhorn cattle [34]. Several CNVs have been identified in cattle for association with milk production traits [35]. Upadhyay *et al*. [34] found genes related to economically important traits of livestock such as *MTHFSD* and *GTF2I* in the CNVRs. Yoshida *et al*. [67] found that the complex BoLA-DRB3 lies in the CNVR associated with Mastitis and Bovine leukemia virus infection in various cattle breeds. Liu *et al*. [45] found the duplication in the *CIITA* gene that showed nematode resistance in Angus cattle. MTHFSD gene covering the CNVR1206 found to be associated with milk protein yield in Spanish HF cattle [68]. Reyer *et al*. [69] found GTF2I in the CNVR1703 region which is associated with feed conversion efficiency in chicken. The *MSH4* gene was found to be associated with impaired gamete formations in laboratory mice and recombination rate in cattle [48, 70, 71]. Brenig *et al*. [72] suggested a dose-dependent effect of Belgian-blue type allele in White Park and Galloway cattle in which uneven pigmented spots were seen in the heterozygous condition, whereas in homozygous condition, there was no pigmentation on the body. Gene Ontology (GO) study revealed that CNVRs are particularly enriched in genes related to immunity, sensory perception, response to external stimuli, and neurodevelopmental processes.

The dominant white coat in sheep is associated with duplication of 190 kb genomic fragment which encompasses three genes viz. the agouti signaling protein (*ASIP*) gene, the itchy E3 ubiquitin protein ligase homolog (mouse) (*ITCH*), and the adenosylhomocysteinase (AHCY) loci [73]. Hillbertz *et al*. [74] identified the duplication in fibroblast growth factor genes and the *ORAOV1* gene in Rhodesian and Thai Ridgeback dogs which is responsible for characteristic dorsal hair ridge. A different pattern of white coat color was reported due to the duplication of the *KIT* gene in pig and in some cattle breeds [75].

### Sheep and goat

GO analysis and functional studies in sheep reported that many CNVRs are associated with genes related to environmental response and biological functions [48]. Liu *et al*. [76] indicated that ZNF family genes mainly expressed in some sheep breeds are involved in regulating evolutionarily divergent biological traits. Higher expression levels of KIF2A and PHKG2 in Gansu Morden sheep breed compared to other sheep breed indicates the association of these genes in disease resistance ability [48]. Yang *et al*. [16] found several important CNV-overlapping genes (*BTG3*, *PTGS1*, and *PSPH*) in diverse sheep breeds which were involved in fetal muscle development, PG synthesis, and bone color.

### Horse

A homozygous deletion in the *AKR1C* gene may be a possible cause of disorders of sexual development such as male-pseudohermaphroditism due to its role in testicular androgen production and sexual development [55]. GO analysis, and functional studies indicated that the equine CNV genes are mainly involved in biological processes and molecular functions related to transmembrane signal transduction, sensory perception, immune response, reproduction, and steroid metabolism. In horse, BMPR1B gene has been reported for its role in the regulation of the rate of ovulation [77]. Ghosh *et al*. [55] confirmed the role of complex CNVR in chr27 involving *CSMD1* gene which encodes for a transmembrane and a candidate tumor suppressor protein [78].

### Chicken

Duplication of segment of DNA at intron 1 non-coding region of the *SOX5* transcription factor interferes with *SOX5* expression, and the regulation of gene expression is critical during cell differentiation for the development of the comb and wattles [79]. Luo *et al*. [80] suggested that CNVs on GGA19 could be a candidate conferring resistance to the Marek’s disease. Late feathering locus in the chicken is due to the partial duplication of the *PRLR* and *SPEF2* genes [81]. Lin *et al*. [82] suggested that *SOX6* gene in chicken also has a similar function as reported in many species for the proliferation and differentiation of skeletal muscle cells. *SOX6* gene expression is positively correlated with the number of CNV for CNP13 region in the chicken genome.

## Conclusion

Recent studies for CNV detection have enabled the construction of CNV map which in turn helps in identification of CNVs associated with economically important traits. With the advancement in the techniques and reduced cost of sequencing, researchers are now focusing on the CNV study for detecting the genetic variations, as CNV shows more inclusions and complex genetic variants than SNP sites. Current research for the identification of the CNV regions (CNVRs) throughout the genome in domestic species will change the concept of breeding for genetic improvement. Development of robust and convenient CNV detection techniques could further facilitate unveiling of genetic secrets for molecular breeding of poultry and other farm animals.

## Author’s Contributions

MP conceptualized and designed the manuscript. BV, SC, and MP prepared manuscript draft and reviewed. CR contributed in literature collection. MP, DRP, and KA edited and made critical comments on the manuscript. MP and BV made critical comments on the revised manuscript and edited for final submission. All authors read and approved the final version.
